# Assessing the digital economy: aims, frameworks, pilots, results, and lessons

**DOI:** 10.1186/s13731-020-00129-1

**Published:** 2020-09-07

**Authors:** Nagy K. Hanna

**Affiliations:** World Bank, Bethesda, MD USA

**Keywords:** Digital economy, Digital development, Digital technologies, Digital innovation, Digital entrepreneurship, Digital strategy, Disruptive technologies

## Abstract

The article discusses the motivations for a holistic assessment of the digital economy. It outlines the pilot assessment program initiated by the World Bank Group and describes the assessment frameworks, tools, and processes deployed in selected pilot countries. It identifies the common challenges faced and lessons learned from applying these assessments in different contexts. These challenges include prioritizing digital diagnosis objectives, addressing inequality and poverty issues, securing participation and partnership of stakeholders, addressing implementation challenges, and integrating digital transformation strategy into a country development strategy. Other challenges include harnessing digital innovation and entrepreneurship, mobilizing local demand for the new technologies, engaging business in digital diagnosis, and adopting multi-disciplinary and whole-of-society approaches. The article addresses the implications of these challenges and draws broad lessons and practical recommendations for developing countries and aid agencies.

## Introduction

A key aim of this article is to provide just-in-time learning from the rich experiences gained from developing digital economy (DE) assessment tools and piloting them in diverse country contexts. Related aims are to share lessons on the effective use of these tools to enhance the quality of diagnostic advice, and improve the data that may be shared and used to inform the advice and subsequent digital strategy formulation. A consensus on a holistic assessment framework would reduce redundancies and confusions that have contributed to higher costs for aid agencies and their client countries. The article also identifies lessons of experience and opportunities to further leverage the proposed assessment framework.

There are many definitions of the digital economy in the literature, reflecting different scopes of relevance, and they are implicit in the assessments undertaken so far by the WBG (Brynolfsson & Kahin, 2000; McAfee & Brynjolfsson (2017). According to the Center for Development Informatics (Bukht & Heeks, 2017), the core of the digital economy is the “digital sector” (ICT) producing foundational goods and services, the “digital economy” consists of the digital sector plus segments of the economy that are essentially digital and do not have analog equivalent, and the widest scope is the use of ICTs in all economic spheres, may be called the “digitalized economy.” In this paper, we *recommend the adoption of the widest scope*, to include the “digitalized” economy. We also define “digital transformation” as the process or ecosystem that transforms all economic spheres and creates the digital economy in its widest scope.

This broad definition of the DE adopted here covers the production and use of digital technologies in both the *private and public* sectors and thus captures the digital dividends for the whole economy. This definition is the most relevant for developing countries and the Sustainable Development Goals (SDGs), since it has been long recognized that most benefits of digital technologies come from its widespread diffusion and use in the economy in the digital and analog worlds (Hanna, 2016; World Bank, 2016). It views the digital economy as an “evolutionary” process that emphasizes the progressive adoption of digital technologies throughout all sectors of the economy.

The article first discusses the motivations for a holistic assessment of the DE. Then, it outlines the pilot assessment program initiated by the World Bank Group (WBG) and defines the methodology used to review DE assessment in various pilot countries. Next, it briefly describes the assessment frameworks, tools, and processes deployed in selected pilot countries. The core part of the article is a summary of the common challenges faced and lessons learned from applying these assessments in different contexts. Finally, it draws key implications and broad recommendations for developing countries and aid agencies.

Over the past few decades, digital technologies have emerged as the most disruptive and transformative forces across all sectors and economies. The speed of disruption, innovation, and diffusion of these technologies is unprecedented, with profound implications for development.

After some doubts and institutional inertia, aid agencies and client countries have become enthusiastic about the digital economy for development. They started to recognize these implications (World Bank, 2016). They are currently aiming to position themselves to help developing countries harness these technologies for development.

Developing countries are increasingly aware of the opportunities of digital dividends and the risks of being left behind in the race of digital transformation.

Currently, there is no standard definition of the digital economy^1^ or standard frameworks and indicators to assess it. Many countries have proceeded with formulating digital economy (or ICT) strategies without the benefit of conducting an objective and holistic assessment upfront. Both countries and aid agencies have also initiated their own programs for digital transformation. Lacking common assessment methodologies and indicators, they risk unmet expectations, and fragmented and poorly coordinated initiatives, with high learning costs.

Harnessing this new enthusiasm will be critical to secure the impact, learning, and sustainability of digital transformation strategies and development cooperation. Rising interest opens up the opportunity to address long-standing gaps among components of the digital transformation ecosystem and between this ecosystem and the country’s development strategy. It calls for new skills and innovations in assessment tools and processes.

This article focuses on a digital economy assessment program, initiated by the World Bank Group (WBG) in 2018. This program provides a leading example of developing a set of diagnostic tools and indicators, integrating and building on WBG research and other aid agencies and think tanks (UNCTAD, 2019; *ITU*, 2013; WEF reviews, etc.), and further piloting and collaboration with several developing countries. The WBG has developed a set of diagnostic tools to assess the digital economy in a holistic way. It provides a potentially powerful framework for addressing complementary reforms and investments in digital infrastructure, skills, innovation, entrepreneurship, governance, policies, institutions, and leadership. These gaps have persisted across developing countries where complementary assets and coordination mechanisms are weak.

## Review methodology

This paper synthesizes a recent review, carried out by the author, and supported by a Digital Partnership Fund, and administered by the World Bank. The review adopted a mixed-methods approach whereby analyses of outcomes are triangulated. The methodology includes in-depth reviews of World Bank reports, literature reviews, and semi-structured interviews with internal stakeholders or assessment teams.

Pilot country assessments covered by this review were advanced enough to generate important lessons. They covered different geographic regions and countries at different levels of development. They adopted different assessment tools and processes. The aim was to maximize the diversity and relevance of engagement experiences and potential lessons, with a modest budget. The purposive sample of assessment pilots covered Russia, Kyrgyzstan, Senegal, Kenya, Lesotho, Tunisia, Morocco, Malaysia, Indonesia, Thailand, and Vietnam (World Bank, 2018a, 2018b, 2019a).

*The overall aim of the review was to provide just-in-time learning.* It took account of emerging new practices, while also drawing on earlier experiences with similar digital transformation strategies, past independent evaluations of digital development projects funded by the World Bank, and the author’s studies of selected countries that have been pursuing digital transformation towards a digital economy (Hanna, 2007, 2011; Hanna, 2016; Hanna & Knight, 2012). The review put as much emphasis on the *process (engagement practices)* adopted as on the data and analyses presented in assessment reports.

Limited by resources, the review does not include interviews with client countries (external) stakeholders, or field surveys.^2^ It is limited by the lack of documentation on the assessment *process* and by the primary focus of peer reviews within the WBG on the final reports, less on capturing the views and changes among local stakeholders.

Drawing lessons as early as possible is a risky but prudent act. The review tried to capture a moving target, to learn about tools and practices that are fast evolving and scaling up to cover many countries. To capture the process of interacting among the stakeholders to produce these assessments, this review used in-depth interviews and engaged participant observers in describing the process.

## Frameworks and indicators

Assessment tools aimed to cover the critical indicators for the main building blocks of the digital economy. They evolved in reaction to country demand and data availability. They were based on an agreed definition of the digital economy. They have in common the recognition of the importance of the digital policies and infrastructures of the economy. They vary however in terms of depth of assessment of the digital foundations, and the extent of their coverage of the transforming sectors of the economy.

The proposed assessment framework covers the digital sector, the digital and non-digital foundations, and, to varying degrees, digital adoption and transformation in the government, private and citizen sectors. Put differently, the assessment framework can be composed of three levels: macro policy foundation (competition, trade, finance, governance, etc.), digital enablers (digital sector, leadership, infrastructure, platforms, policies, skills, finance), and sectoral transformation (vertical ICT applications in key economic sectors, like public services, education, and agriculture). It could be complemented by a modular assessment of key components or subcomponents like digital commerce, digital finance, and digital platforms.

These components and their interactions co-produce the digital dividends or economic impact of digital transformation.^3^ Much of the literature has focused on one or two components of the digital economy ecosystem, such as internet penetration or indicators of the innovation subsystem, but rarely if any on the whole ecosystem and its interactions with the rest of the economy (World Bank, 2016 and 1999; Hanna, 2017, Hanna, 2016 and Hanna, 2009; Foster & Heeks, 2013; Heeks, 2014; Carr, 2004; Dutta & Mia, 2008; Stiglitz & Greenwold, 2014).

**Fig. 1:**
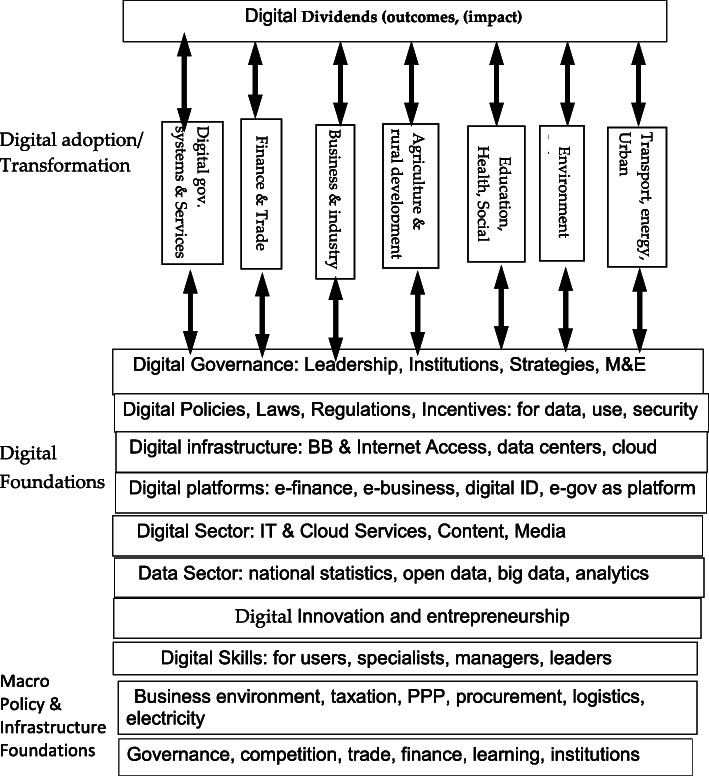
Digital economy ecosystem

Each of these building blocks (or levels) is characterized by a set of indicators. These are used to determine the relative strengths and weakness of the foundations of the digital economy, and the degree of diffusion of digital transformation in various economic sectors of a country. Figure 1 depicts this holistic framework.

Some assessment methodologies have focused on a subset of the interdependent elements of the digital economy ecosystem, for example, UNCTAD’s Rapid e-Trade Readiness Assessment. A pilot assessment of Malaysia’s digital economy (World Bank, 2018a, 2018b) focused on e-commerce, covering digital connectivity, digital entrepreneurship, digital payments, and taxation of the digital economy. Another example of focused assessment is the Digital Government Readiness Assessment (DGRA) which focused on the public sector transformation and service delivery. A focused approach can give more in-depth attention to the components and interactions of a subset of the digital economy (such as e-commerce, innovation, or government services). But it may miss some of the shared foundations and synergies that would be captured by the above more comprehensive framework.

## Assessment process

As pioneering activities, the pilot assessments under review did not benefit from explicit guidelines or established practices for using the digital economy assessment tools. They operated under significant process uncertainties. Assessment teams felt this gap, even while enjoying the flexibility to improvise and innovate to carry out the task.

Collecting data and making estimates used a variety of methods: online surveys, expert panels, face-to-face interviews with the representative of stakeholders, and desk research drawing on international databases. Some pilots relied mainly on online surveys and minimal investment in engaging experts and stakeholders. Others involved broad consultations and knowledge-sharing events. Upfront national workshops involving international experts and representatives of local stakeholders proved valuable to reach a shared understanding of the whole ecosystem (Fig. 1) and its indicators. Concluding workshops were also critical to improve the validity of the results and help participants move to the next steps of strategy formulation and implementation.

Assessment processes were strongly influenced by country capacity and prior engagements between the WBG and client country. They were also shaped by the skill composition of the WBG team. Pilot assessments in more advanced countries like Malaysia and Russia tended to rely more on collaboration with local anchor institutions. For less advanced countries, the assessment process involved more data collection, expert surveys, and educational workshops. The most valued aspects of the process were assessment workshops that engaged global and country experts from the most advanced transforming economies such as South Korea, Singapore, and Estonia. Best assessment practices also involved multi-level communications with top policy makers as well as focused target groups and those concerned with digital strategy formulation and implementation. They put more emphasis on interactive processes and building coalitions for policy reforms and digital transformation capacity among implementing institutions, rather than data collection and reports.

## Early results

Most tangible results within the WBG were (1) increase collaboration across global practices to build a comprehensive status of a country’s digital economy, (2) engage new stakeholders beyond the ministries concerned with ICT, (3) focus attention on policy and strategic issues facing the digital economy, (4) increase the objectivity of country assessment and the realism of target setting, and (5) provide the basis for preparing a national investment program for digital transformation. It is too early to judge whether these holistic assessments have triggered major changes in stakeholders’ behavior in client country and/or the aid agency?

Early results of assessments were influenced by several factors. The scope of the assessment framework and indicators used in various pilots was limited by the scarcity of quantitative benchmarking data. In some pilots, this may have distorted the priority of topics to be covered. This issue is most critical for developing countries where standard indicator data on key dimensions of the digital economy are likely to be in short and unreliable supply. National statistics offices are lagging behind in capturing the data necessary to assess the emerging digital economy. Several country assessments could not generate the missing data, given time, and resource constraints.

Limited availability of comparable data for benchmarking put significant constraints on analyzing key issues such as the digital divide, and the interactions between the local digital sector and the use of digital technologies to transform various economic sectors. For example, current benchmark data were too aggregate to differentiate digital access and adoption among large, medium, and small enterprises, among household income and education levels, and between advanced and lagging regions. Hence, the analysis of the level and distribution of social and economic impact left much room for improvement. Similarly, benchmarking data on the digital sector (digital sector as a percentage of GDP, Jobs, etc.) is too aggregate to capture potential synergies between software (and systems integration) services and local capabilities available to transform adopting sectors.

Even when benchmarks were generated from international tables and expert judgments for the pilot countries, these data were not always interpreted, analyzed, and effectively used for strategy formulation. Some of the explanations should come from the discussions among the experts who made judgments to arrive at these benchmarks. This would make the recommended policies more evidence-based.

The impact of assessment tools has been strongly conditioned by the skills of the country and aid agency teams, by the process used to carry out these assessments, and by the skills of ultimate users of assessments. The best assessment frameworks and tools cannot substitute for the skills and experience of the participants. Much of the value added of digital assessment instruments depends on organizational issues facing the users of these tools. Assessments should pinpoint the gaps and measures to collect better data for future assessments. They may also need to look beyond traditional sources of data, from both public and private players. When benchmarking data is generated from “expert judgments,” the process used to generate such qualitative judgments should be made transparent.

## Challenges and lessons learned

Experience from developing and applying digital economy assessments methodologies points to the following lessons and challenges: 4

1. Clarifying and prioritizing objectives
2. Securing coherence among assessment tools
3. Addressing poverty and inequality
4. Attending to process, participation, and partnerships
5. Strengthening country implementation
6. Integrating innovation
7. Integrating digital economy into a country development strategy
8. Promoting local demand and effective use
9. Collaborating across sectors and practices
10. Engaging business
11. Managing increasing demand and risks

### Clarifying and prioritizing objectives

In most pilots, the primary objective of assessment became data collection. Almost all resources went to tool refinement and data improvement; little was left to formulating the new or updating the ongoing digital development strategy. Assessment data was at times confused with strategy.

Pilot assessments aimed at different implicit objectives, ranging from tool development and data collection, to building capabilities for assessment, generating national consensus on strengths and weaknesses, and/or formulating specific recommendations and designing digital transformation strategy. It is critical to clarify these objectives upfront and to prioritize them based on client needs. The balance among competing objectives has implications for the tools and processes used for assessment, for the engagement team skill mix, resources, and accountability.

### Securing coherence among assessment tools

Drawing on pilot assessment experiences within the WBG, coherence among various digital economy (DE) assessment tools, proved to be a key challenge. Various global practices^5^ and regions became attached to their own assessment tools. The original WBG goal was to adopt rapid prototyping and move towards a standard comprehensive assessment framework that would be adapted only as deemed necessary to specific country conditions.

Determining the boundaries of the digital economy ecosystem to be diagnosed is a critical decision. A comprehensive coverage of the ecosystem (Fig. 1) would capture the key interdependencies within the ecosystem and enhance the economic impact^6^. But the scope of assessment may be dictated by the time, skills, data, and other resources that may be available to the assessment. Country leadership may be most interested in specific aspects of the digital economy and that may help determine the focus of assessment tool.^7^

Consensus on a comprehensive yet modular assessment framework would reduce assessment costs, provide clear guidance to assessment teams, allow for sequential assessments, and facilitate cross-country learning*.* That would require close cooperation among the concerned global practices and regions, and across aid agencies.

### Addressing poverty and inequality

Unless harnessed for inclusive development, digital technologies are likely to contribute to rising inequality. Evidence so far shows that the aggregate impact of digital technologies is highly uneven among and within developing countries (World Bank, 2016). Yet, many of these technologies, such as mobile money, offer new and significant opportunities to reduce poverty and achieve shared prosperity. However, none of the sample pilot countries made moderating inequality and reducing poverty a central focus for their digital economy strategy.

The current assessment tools did not provide adequate coverage of digital inclusion and income inequality at the national and subnational levels. Current national-level assessment indicators are too aggregate to capture digital-related income, gender, and geographic disparities. Assessments often failed to explain the persistence of barriers to inclusion: what explains slow and uneven adoption? How effective is current usage in contributing to poverty reduction? Why promising applications for poverty reduction fail to scale up? What mechanisms would be needed to counter monopolistic and clustering tendencies of digital platforms and digital industries? Assessments did not attempt to systematically track the distributional and empowerment impact of the new technologies.

Concern about poverty and inclusion has implications for both what is assessed, and how the assessments are carried out. Assessment tools should be accessible to all stakeholders so they would not be limited to a dialog of the elite. Pilot country assessments did not devote much attention to empowering representatives of poor communities and social intermediaries to participate beyond the supply of data. Assessment results were not used to engage poor communities in shaping and implementing an inclusive transformation strategy.

### Attending to process, participation, and partnership

The process used to assess the DE can influence outputs, outcomes, impact, and accountability. It may be driven by such objectives as promoting client participation and ownership, forming coalitions and partnerships, developing capacity and institutions, and mobilizing local knowledge.

Pilots varied in the process used to apply assessment tools. Excessive attention was given to refining the tools, data collection, and reporting but often at the expense of engendering successful ownership and effective use of the results. The degree of local stakeholder participation in DE assessment and downstream strategy development varied greatly. Little effort was made to influence the composition of the local participating team to include intermediary institutions representing small business, civil society, trade and professional associations, and poor communities. Pilots could have benefitted from upfront stakeholder analysis to guide participation and collaboration within the country.

Local anchor institution(s) were critical to securing a responsive, client-driven assessment process. The degree of securing effective and inclusive participation was influenced by the designation of a capable anchor institution and its own capacity to engage key stakeholders. Few pilots sought to secure broad and balanced participation. Fewer were involved in mobilizing underrepresented groups and engaging social intermediaries.

### Strengthening country implementation

Most pilots did not assess the implementation quality of past strategies nor used past performance to render judgment on the capabilities of existing institutions to implement proposed strategies. Yet, country experience suggests that implementation of DE strategies is the hardest part of digital transformation (Hanna, 2016; Hanna & Knight, 2011). Successful countries have done the most preparation for the implementation stage during strategy formulation. Digital transformation calls for developing new institutions, mobilizing the local ICT services sector, strengthening digital governance, and creating new cadres of digital leadership, including chief information and innovation officers (CIOs).

### Integrating innovation

Pilot country assessment of the DE focused on the adoption of the latest technologies. It neglected to include incremental, adaptive, and bottom-up innovation that would be necessary for the diffusion of existing technologies and their fit into new contexts. Assessments of local innovation and entrepreneurship ecosystems did not give due attention to local best practices within the public and private sectors that could be scaled up and integrated into a national DE strategy.

The rise of the digital economy calls for unconventional economic thinking and policy innovations. It calls for exploring new pathways to local value creation and capture. For example, servicing local markets and poor communities would often require creating blended digital-analog processes. Assessment should push for innovations that come from the grassroots, cross-sectoral collaboration, and beneficiary engagement (Hanna and Picciotto, 2002; McAfee & Brynjolfsson, 2017; Foster & Heeks, 2013; Hanna, 2011; Mazzucato, 2013; World Economic Forum, 2012).

### Integrating into a country development strategy

One key finding of this review is that digital diagnostic tools made only modest progress in narrowing the gap between country development strategies and digital economy strategies. Digital diagnostics are often conducted in isolation of country economic development diagnostics and thus fail to make clear the connection between progress on digitalization of the economy and progress towards achieving the Sustainable Development Goals (SDGs). Ideally, the formulation of both the digital economy and country economic strategies should proceed interactively, as digital technologies can offer new options for development strategies, while development strategies may harness digital technologies for new uses and innovations.

The present gap between digital development practice and country economic development practice should be bridged. A diagnostic tool, on its own, cannot bridge this gap. Pilot assessments that succeeded have fully engaged the core ministries concerned with finance and economy. More progress will depend on addressing the underlying institutional barriers that perpetuate the gap between development and technology specialists in developing countries and aid agencies. Aid agencies and countries’ planning agencies have developed their own processes and routines for investment planning and country programming. These routines tend to reinforce silo thinking. They have not been adapted to take account of the digital revolution. Yet, digital diagnostics can facilitate strategic thinking about the use of digital technologies for deep economic transformation.

### Promoting local demand and effective use

Assessment indicators did not adequately capture actual adoption and effective use of digital technologies in general, and in public agencies, small businesses, and traditional industries, in particular. Yet, the greatest dividends are ultimately realized from diffusion and spillover of digital technologies into key economic sectors.

For most developing countries, there is significant scope to stimulate public demand for innovative and locally tested digital solutions, especially those coming from local innovators and technology SMEs. Despite significant strides in providing citizens with government services online, the uptake is still relatively low. This suggests the need for demand mobilization measures, such as strengthening demand for good government, retraining civil servants, and promoting digital and media literacy.

### Collaborating across sectors and practices

Advancing economy-wide digital transformation requires a whole-of-government approach within countries and multi-disciplinary development practices within aid agencies. A core objective of a holistic assessment of the DE is to provide a cross-sectoral view of the state of the digital economy and thus enable the country to design coherent policies and programs, and coordinate aid and investment for digital transformation. Economy-wide DE assessment is expected to improve collaboration among economic sectors and development practices to deliver more integrated solutions to advance digital transformation and help countries break their own ministerial and sectoral silos.

The author’s review suggests some modest progress is being made. But gaps between sectoral practices persist in the WBG and within pilot countries. Having common assessment tools is a good step. But the current operating model within the WBG (and other aid agencies) tends to inhibit collaboration and cause fragmentation, internal competition, and budget “dogfights” (IEG, 2019). In most pilots, collaboration was limited to dividing up different components of the DE assessment to corresponding sectoral practices, working in parallel. This risked having siloed assessments and less coherent strategies.

### Engaging business

Engaging business as an equal partner in shaping national DE strategy remains a key challenge for developing countries and aid agencies. While significant progress has also been made to secure collaboration between the World Bank and its private sector arm, much remains to be done to engage IFC in the full cycle of assessment, strategy formulation and implementation, and downstream investments. Full IFC engagement in the digital economy would require that WBG prioritize upstream policy reforms that unlock opportunities for the deployment of private-sector solutions in the DE. It would also require prioritizing investments in the local digital businesses that can strategically contribute to the whole digital economy ecosystem. It would require aligning incentives for staff to reward collaboration in digital economy assessment and strategy implementation. That would require a cultural shift from tactical to a strategic partnership between the World Bank and IFC.

Similar challenges have been experienced in securing collaboration among government agencies, the digital sector, and user businesses in the pilot country. Effective engagement of the private sector in DE assessment has been constrained by the presence of local monopolies, weak representation of small business, mistrust in government, and limited understanding of the assessment methodology, among others.

### Managing demand and risks

On the whole, the diagnosis of pilot digital economies erred more on strengths and opportunities, less on the accompanying risks, tradeoffs, and downsides of digitization, and the country’s capacity for managing these risks. Insufficient attention has been paid to ways by which digital platform firms exacerbate income inequality and adversely impact the distribution of the gains. Assessments may give special attention to the development of local digital platform firms that can serve local needs and capture value and digital intelligence from local data.^8^ Assessment may cover policies and innovations to promote digital upgrading (value addition in the data value chain) and to enhance domestic capabilities to refine local data (UNCTAD, 2019; World Bank, 2019b). Promoting digital innovations for the data economy also raises the risks to data privacy and security, and the importance of building policy making capabilities for harnessing digital data. It is also critical for developing countries in particular to use the diagnosis to assess the disparate impact of digital innovations and indiscriminate use of disruptive technologies on semi-skilled jobs, and local capacity to create alternative jobs and skills.

After a slow response to the digital revolution by countries and aid agencies, spanning several decades, there are currently increasing demands for financial and technical assistance from governments in many developing economies. This recent increase may be due to growing awareness within countries and aid agencies concerning the centrality of digital transformation for economic development. Other factors augmenting such demand is the current global COVID-19 pandemic, global economic recession, demonstration effects form pioneering countries, and growing maturity and research in a digital economy. Most aid agencies are not yet fully equipped to fully respond to this apparent sudden explosion in demand, with adequate skills and processes to manage their fiduciary and safeguard responsibilities, and associated reputational risks. Many developing countries are also not adequately ready to manage the risks associated with heavy dependence on core digital infrastructures, strategic systems, strategic data, and associated policies to promote trust and digital resilience.

### Implications for developing countries and aid agencies

The experiences drawn from the digital economy assessment program lead to five broad implications and recommendation areas for both developing countries and aid agencies: (1) develop digital *leadership* in countries and aid agencies, (2) build the necessary *skills* within the country and aid agency to help clients harness the digital revolution, (3) develop and disseminate the emerging digital assessment *tools* while attending to the *process* of using and implementing them effectively, (4) address *poverty and inequality* challenges as an integral part of assessments, and (5) create a country- and agency-wide knowledge sharing and *learning platform*.

*First, digital leadership and institutions*: Key elements of the digital transformation ecosystem reside in different parts of the aid agency and in the country. A holistic approach to a digital economy would demand ecosystem-wide leadership and coordination (Hanna, 2018; Hanna and Qiang, 2009). This has posed a major challenge to collaboration and integration in the pilot countries, and under the current WBG’s operating model. However, the digital economy approach, appropriately applied, may provide an opportunity for the country and aid leadership to bring coherence to digital transformation initiatives and encourage collaboration and shared learning.

It is critical to harness investments in sectoral applications and the digital foundations to be mutually supportive, and to support the broader development agenda. Ministries in charge of developing the digital foundations should aim to facilitate key sectors like education, health, finance, trade, and agriculture. The sectoral transformations would require policy and institutional investments that would build on these foundations. Leaders should communicate their vision of how the digital economy would contribute to inclusive growth and poverty reduction; allocate the necessary budgetary resources to develop the tools, skills, staffing, partnerships, and organizational learning; and ensure that digital economy strategies are integrated into country development strategies.

Digital economy assessment should aim to mobilize the necessary country-level leadership and digital leadership institutions. Assessments should conduct institutional and stakeholder analyses to engage public agencies, and business and civil society organizations in a co-leadership of the digital economy. Assessments should aim to induce country-based coordination and policy coherence among major aid agencies and sources of finance for the DE.

Second, *Skills and staffing*: Building a cadre of aid agency and civil servants with the appropriate mix of skills is perhaps the biggest challenge of practicing digital transformation. Several means are worth exploring: outsourcing, secondment, building knowledge partnerships with more advanced countries, and retaining a global network of trusted advisors.

Third, *Frameworks and Processes*: Country objectives for launching digital economy assessment should be clarified in order to identify quick wins, set realistic expectations, and build the digital foundations for the long term. Country objectives should determine the appropriate scope and duration of engagement, and the necessary budget, skills, and partnerships. Assessment tools and processes should be designed to support the broader digital economy strategy formulation and implementation process.

Long-term assessment engagements are recommended, to cover all stages of digital strategy formulation and implementation and maximize impact and learning. At times, a quick and timely assessment may be preferred but should be viewed within a sustained strategy of institution building. Excessive short termism would run the risk of focusing assessment attention on the hard infrastructure rather than the whole digital transformation ecosystem, including digital policies, institutions, and capabilities.

Assessments should cover country implementation mechanisms. Whenever possible, assessment should be led by a local anchor institution capable of engaging key local actors. Countries and their development partners should think through the implementation process during the early assessment phase and throughout strategy formulation. Stakeholder, political economy, and institutional analyses are key to the implementation phase. Building state capacity to regulate the digital economy is increasingly critical to competition, inclusion, and security. Similarly is state capacity to manage digital disruption and resilience.

To deal with the novel policies and institutions of transformation, governments and aid agencies need to change their processes and tools to become more agile and client-focused. This applies to ICT procurement and financing instruments in particular.

Fourth, *poverty reduction and inequality*: The experience of developing countries suggests that digital transformation strategies should be designed to be inclusive (Hanna, 2010; Hanna, 2011). An inclusive DE requires persistent efforts and innovative measures to address non-digital complementary factors (analog complements) and to overcome deep-rooted contributors to the digital divide (Hanna, 2017; World Bank, 2016).

Adoption, diffusion, and effective use of digital technologies are critical to inclusive growth and poverty reduction. Digital diagnosis should therefore assess demand arising from lagging traditional sectors, small businesses, and poor communities. Due attention should be given to public demand for digital adoption, and effective use in the social sectors like health and education. Governments need to partner with the private sector, trade and professional associations, and social intermediaries to invest in digital literacy, digital public services, local content, societal applications, and other measures of demand mobilization. Assessments should capture these sectoral needs and opportunities.

Attention should be given to jobs, gender, governance, environment, and other cross-cutting issues impacting shared prosperity. Transformation leaders should monitor digital economy diagnostics and follow-up assistance to ensure that inclusion challenges are systematically addressed, and digital transformation is harnessed to reduce poverty and moderate income and regional inequalities. Assessments should cover monitoring and evaluation of local innovation programs that are most relevant to inclusion. Assessment may give high priority to the use of digital identification as a platform for inclusion.

*Fifth, learning and knowledge sharing*: Managing digital transformation demands accelerated social and institutional learning. Assistance to countries to pursue digital transformation demands agile, intensive, and collaborative learning. The piloting approach to DE assessment taken by WBG is most appropriate for such learning. However, several factors are likely to slow such learning, including the lack of learning and knowledge sharing platforms for digital assessments and transformation.

Assessment tools, processes, and reports should be shared widely within among aid agencies and countries. At this stage, the lack of a standard assessment methodology should leave space for debate, learning, and creativity. Partnerships with local universities and think tanks should be encouraged, to pilot and institutionalize assessment tools, and with international organizations to improve the effectiveness of these tools and facilitate donor coordination.

The assessment program should create a culture of collaboration, risk-taking, learning from mistakes, openness, and trust. Aid agencies should view assessments as opportunities for dialogue, research, and learning with clients. Creating shared platforms would leverage the considerable economies of scale in collecting data and applying analytics to digital assessment.

## Conclusions

Developing countries are under immense pressure due to the current pandemic and global economic recession, and many are already heavily indebted and facing climate change disruptions. Diagnosing the DE, prioritizing policy and institutional reforms, and building the digital enablers have become more critical than ever, particularly in poor and heavily indebted countries. The current situation calls for quick responses, fast learning, and innovative and holistic solutions to help countries accelerate their digital transformation. Yet, building digital economies is a marathon, not a sprint. This means sustained engagement from local leaders and aid agencies.

This review identified significant opportunities for countries and aid agencies to learn to diagnose the emerging digital economies, develop coherent digital transformation strategies, and devise whole-of-government and whole ecosystem implementation mechanisms. Countries may view the opportunities beyond these multiple crises to address what future do they want and to harness the digital revolution to realize the promising dividends in areas made clear by these crises. They may harness their digital economy to reduce poverty and inequality and increase their economic, environmental, and digital resilience.

Lessons from the digital diagnostic program would reinforce key messages: develop digital leadership and institutions; strengthen both digital and non-digital foundations of the DE; align DE strategy to support overall country development strategies; set sectoral transformation priorities in health, education, and essential public services; develop digital economy skills and capabilities; address poverty and inequality indicators early on at the diagnostic stage; engage underrepresented stakeholders and SMEs; support digital innovation and entrepreneurship to underpin adaptive-learning transformation strategies; mobilize demand and widespread adoption of promising applications and local innovations; and build platforms and culture for innovation and leaning. These suggestions are as applicable for aid agencies like the World Bank, as they are for developing countries.

## Data Availability

Not applicable

## Abbreviations

DE: Digital economy
BB: BroadBand
IT: Information technology
ICT: Information and communications technology
GDP: Gross domestic product
DGRA: Digital Government Readiness Assessment
SMEs: Small and medium-sized enterprises
WBG: World Bank Group
WEF: World Economic Forum
UNCTAD: United Nations Commission on Trade and Development
ITU: International Telecommunications Union
PPP: Public-private partnership
e-ID: Digital identification

## Footnotes

1 Digital economy is used here interchangeably with other popular terms: information, innovation, learning, and knowledge economy. Attempts to define and measure each tend to emphasize key aspects or components of what we call here the digital economy.

2 The Independent Evaluation Group (IEG) of the World Bank has launched an evaluation of digital economy initiatives in the context of a broader report on World Bank Group effectiveness in assisting countries in responding to disruptive and transformative technologies, which draws on and complements this review.

3 Several recent attempts have been made to define the digital ecosystem’s components, their interrelationships, and their economic (GDP) impact. For example, Katz/ITU constructed a digital ecosystem development index composed of 64 indicators (factors) and applied econometrics to measure their economic development impact (digital dividends). These various attempts provide useful insights. But they suffer many shortcomings as they do not capture the complementarity among these factors and the socio-economic context of the country. This paper does not attempt to measure such economic impact.

4 Some of these challenges and lessons are applicable to development practices more generally, but are most relevant to digital development practice in particular Independent Evaluation Group, World Bank, 2015 and 2019; Hanna, 2018; Hanna & Knight, 2011 and 2012).

5 The World Bank is organized along sectoral practices such as infrastructure (covering energy, transport, and digital development); human development (covering education, health, etc.); finance, competition, and innovation; governance, macroeconomic management, trade, and investment; and so on, covering all geographic regions. Most aid agencies are similarly organized along sectoral or disciplinary practices.

6 For a pilot comprehensive assessment, see World Bank. (2018a). *Competing in the Digital Age: Policy Implications for the Russian Federation*.

7 For a pilot focused assessment see the *Malaysia’s Digital Economy* assessment covered only those components most directly impacting digital commerce (World Bank, 2018b). An assessment methodology, tailored for Africa, has focused on the digital foundations for the economy; for example, *Nigeria Digital Economy Diagnostic Report* (World Bank, 2019a).

8 Countries at all levels of development risk becoming mere providers of their row data to the few global platform firms (Microsoft, Apple, Amazon, Google, Facebook, Tencent, and Alibaba), while having to pay for the digital intelligence produced with those data by the platform owners (UNCTAD, 2019).
